# Gallbladder perforation without cholecystitis in a patient awaiting liver transplantation: a peculiar case report of anaemia in cirrhosis

**DOI:** 10.1186/s12876-019-1018-9

**Published:** 2019-06-27

**Authors:** Marco Biolato, Claudia Tarli, Giuseppe Marrone, Brunella Barbaro, Antonio Liguori, Antonio Gasbarrini, Antonio Grieco

**Affiliations:** 1 Fondazione Policlinico Universitario A. Gemelli IRCCS, Roma, Italy; 2 Università Cattolica del Sacro Cuore, Roma, Italy

**Keywords:** Liver transplant, Hemoperitoneum, Anaemia, Gallstones, Ascites, Case report

## Abstract

**Background:**

Acute anaemia in decompensated liver cirrhosis is commonly caused due to gastrointestinal bleeding; however, sometimes, detecting the site of blood loss is challenging.

**Case summary:**

A patient on waitlist for orthotopic liver transplantation because of decompensated liver cirrhosis was admitted with acute anaemia and recurrence of ascites. Their abdomen CT showed migration of gallbladder stones in the pelvis while paracentesis documented hemoperitoneum. A diagnosis of gallbladder perforation was performed.

**Conclusion:**

Challenging choice of a “wait and see” strategy with conservative therapy, avoiding high-risk cholecystectomy, resulted in a successful liver transplant.

## Background

In advanced liver cirrhosis, a haemorrhage is usually secondary to complications of portal hypertension, such as gastroesophageal variceal rupture, hypertensive gastropathy, gastric antral vascular ectasia or peptic ulcers [1, 2]. Acute gastrointestinal bleeding is mostly evident clinically; however, sometimes, the site of bleeding is not easy to detect. Gallbladder perforation occurs in 2–15% of acute cholecystitis cases with or without gallbladder stones while haemorrhagic cholecystitis and massive hemoperitoneum are very rare [3]. Niemeier, in 1934, classified gallbladder perforations into three types: type I (acute) was associated with generalised biliary peritonitis, type II (subacute) with pericholecystic abscess and localised peritonitis and type III (chronic) with internal or external fistulae [4]. Cholecystectomy is the treatment of choice; however, in cases of high post-operative risk, endoscopic transpapillary gallbladder stent placement is proposed [5]. The presence of gallbladder perforation without cholecystitis was not reported in literature. This study reports the case of a patient with advanced liver cirrhosis awaiting liver transplantation, who had presented with unexplained acute anaemia and received a final diagnosis of gallbladder perforation without cholecystitis, successfully treated by conservative treatment.

## Case presentation

**Fig. 1:**
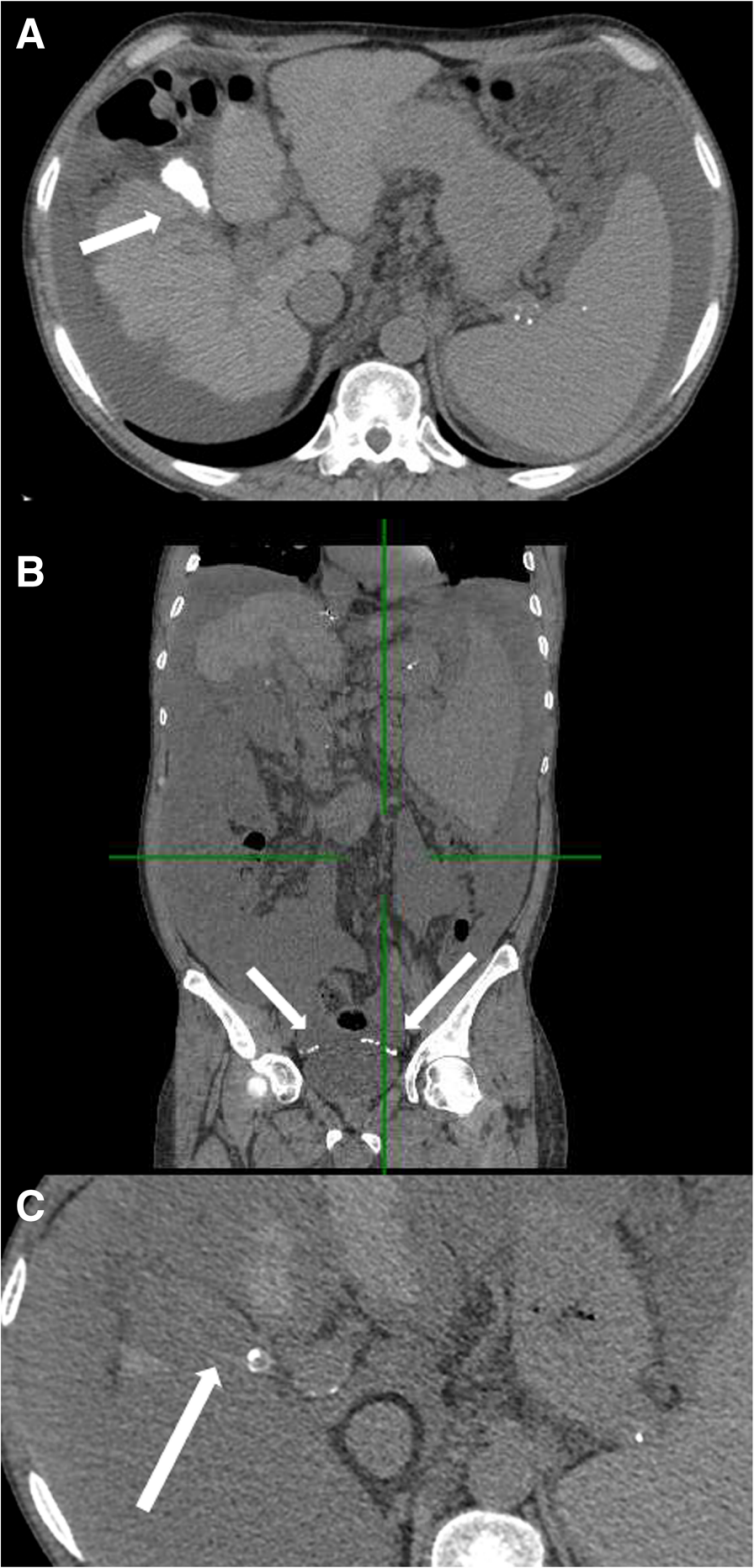
**a** Abdominal CT performed a month before: scleroatrophic cholecystitis whose lumen is completely occupied by lithiasic formations (arrow). **b** Abdominal CT performed in urgency: numerous radiopaque elements were evident in the pelvis (arrows) alongside dense ascites. **c** Abdominal CT performed in urgency: scleroatrophic gallbladder bearing only one stone inside and irregularity along the wall but no frank interruption

A 55-year-old man was on the waiting list for orthotopic liver transplant because of a decompensated HBV-related liver cirrhosis (Child C11, MELD 16) complicated by intractable ascites for which a transjugular intrahepatic portosystemic shunt (TIPS) had been placed 5 months earlier. He was also affected by asymptomatic gallbladder stones and admitted to our unit because of sudden recurrence of abdominal distension. The patient reported a few days before severe epigastric pain that lasted for about 20 min and was spontaneously resolved; he, however, denied hematemesis or melena. At admission, physical examination revealed normal vital signs. Laboratory workup revealed a haemoglobin level of 6.4 g/dl (compared to that of 10.3 g/dl recorded 3 weeks earlier), leucocyte count of 4930/mm3, platelet count of 49/mm3, international normalised ratio of 1.44, bilirubin 3.2 mg/dl, alanine aminotransferase 25 UI/l, aspartate aminotransferase 47 UI/l, alkaline phosphatase 105 UI/l, gamma glutamyl transferase 27 UI/l, albumin 2.0 g/dl, creatinine 0.91 mg/dl and C reactive protein 48 mg/l. The patient was made to undergo urgent blood transfusion. An urgent gastroscopy revealed no varices, gastric ulcer or any other source of bleeding. An ultrasound sonography confirmed the presence of ascites and normal flow within the portal vein and the TIPS. The patient underwent diagnostic paracentesis with leakage of high-pressure blood-like fluid: the cell count analysis confirmed hemoperitoneum (haemoglobin 2.8 g/dl) and showed normal leucocyte count and no malignant cells. The analysis of the ascitic fluid also documented a transudate (total proteins < 2 g/dl, LDH 162 UI/l) with high serum-ascites albumin gradient (1.4 g/dl), bilirubin concentration of 2.9 mg/dl and ascitic culture as negative. A contrast-enhanced abdomen CT-scan showed a scleroatrophic gallbladder bearing only one stone inside and irregularity along the wall, but no frank interruption while numerous radiopaque elements were evident in the pelvis alongside dense ascites (Hounsfield Units 19; uncomplicated ascites normal value 0–15) [6], configuring a picture like “starry sky” (Fig. 1). No ectopic gallbladder varices have been recorded. Both abdominal CT and ultrasound performed a month before described contracted gallbladder, whose lumen was completely occupied by stones. A perforation of the gallbladder with occult bleeding was therefore diagnosed. Based on absence of fever, signs of peritoneal involvement, stability of haemoglobin levels and expected waitlist time, a conservative therapeutic strategy (antibiotic therapy and control of the coagulation parameters, haemoglobin and renal function) was chosen to avoid high-risk cholecystectomy before liver transplantation. In the following days, clinical and laboratory parameters remained stable and, particularly, no fever or exacerbation of hepatic encephalopathy were observed. The patient was discharged in 15 days, asymptomatic, with a stable level of haemoglobin. Two months later, the patient underwent a successful liver transplantation. During the transplantation, the surgeon visualised the stones that had migrated into the pelvis and decided not to remove them because of an unfavourable risk-benefit ratio. The pathological report documented a scleroatrophic gallbladder with chronic inflammation but did not identify any perforation.

## Discussion and conclusions

This study reported a peculiar case of gallbladder perforation without cholecystitis in a cirrhotic patient awaiting liver transplantation. The most interesting elements of this case are the diagnostic work-up, the presence of an advanced liver disease in the patient and the modality of treatment of one of the most severe complications of biliary pathology.

For patients with liver cirrhosis, causes of acute anaemia include haemolytic anaemia (autoimmune or secondary to hypersplenism), aplastic anaemia or occult haemorrhage [7]. Upper gastrointestinal bleeding in some cases does not occur with haematemesis or melena and may be due to variceal haemorrhage, portal hypertensive gastropathy, gastric vascular ectasia, peptic ulcer, Dieulafoy’s lesion, Mallory-Weiss syndrome or portal hypertensive enteropathy [8]. Another possibility can be hemoperitoneum due to spontaneous ruptured intraperitoneal varix or hepatocellular carcinoma [9, 10]; in these cases, diagnostic paracentesis with ascitic fluid cytology confirms the diagnosis [11].

Gallbladder perforation is a rare complication of acute cholecystitis: Derici and coll. reported a prevalence of 4.8% in a retrospective study including 332 patients [12]. In these cases, gallbladder perforations are attributed to ischemia and necrosis secondary to inflammation and are usually located at the fundus which, as a part of the gallbladder, is most distant from the organ’s blood supply [13]. Hemoperitoneum associated with gallbladder perforation is a rare event and has been associated with haemophilia, diabetes, abdominal trauma or anticoagulant therapy [14–18]. Diagnosis of gallbladder perforation is a difficult challenge in cirrhotic patients due to concomitant edema of gallbladder wall, leukopenia secondary to hypersplenism and ascites [19, 20]. Stones outside the gallbladder documented at the CT scan are considered a direct sign of the perforation [21, 22]. In our patient, gallbladder morphology changed from swelling to scleroatrophic compared to previous CT exams, whereas no thickened walls of gallbladder, signs of interruptions, pericholecystic inflammation or liver abscesses were documented [23, 24].

Cholecystectomy is the preferred option for treatment of symptomatic gallbladder stones, whereas gallbladder perforation represents a surgical urgency [25]. Mortality rate for type I gallbladder perforation in the series of Gunasekaran, which did not include cirrhotic patients, was 28%. Data on mortality rates of type I gallbladder perforation in cirrhotic patients are not available in literature; however, they are probably even higher because of risks of liver decompensation or sepsis. However, cirrhotic patients have a higher rate of intraoperative and postoperative complications, especially postoperative infections [26, 27]. In these patients, MELD score is a progressive and independent predictor of morbidity and mortality [28]. In this cirrhotic patient with MELD 16, the mortality rate after open cholecystectomy for uncomplicated acute cholecystitis was found to be about 10% according to Dolejs experience. Furthermore, an open cholecystectomy would have increased about 10–15% of the mortality rate of the subsequent liver transplant. The indication for surgery therefore needs to be weighed against the risk of performing any surgery on cirrhotic patients. Non-surgical therapeutic approaches include percutaneous ultrasound-guided gallbladder drainage [29] and endoscopic transpapillary gallbladder stent placement [30]. This study’s patient had advanced cirrhosis and was awaiting liver transplantation; based on an absence of peritonitis, clinical stability and expected waitlist time, we aimed for a conservative strategy to spare any surgery before the liver transplant. Clinical stability, a careful clinical observation and monitoring of laboratory tests facilitated a “wait and see” strategy, resulting in a successful liver transplant.

## Data Availability

Data sharing is not applicable to this article as no datasets were generated or analysed during the current study.

## Abbreviations

CT: Computed tomography
HBV: Hepatitis B Virus
LDH: Lactate Dehydrogenase
MELD: Model for end-stage liver disease
TIPS: Transjugular intrahepatic portosystemic shunt
